# Patient feedback to improve quality of patient-centred care in public hospitals: a systematic review of the evidence

**DOI:** 10.1186/s12913-020-05383-3

**Published:** 2020-06-11

**Authors:** Eunice Wong, Felix Mavondo, Jane Fisher

**Affiliations:** 1 BehaviourWorks Australia, Monash Sustainable Development Institute, Monash University, Melbourne, Australia; 2 School of Public Health and Preventive Medicine, Monash University, Melbourne, Australia; 3 Department of Marketing, Monash University, Melbourne, Australia

**Keywords:** Patient feedback, Patient experience, Patient-centred care, Quality improvement, Quality of care, Public hospitals

## Abstract

**Background:**

To review systematically the published literature relating to interventions informed by patient feedback for improvement to quality of care in hospital settings.

**Methods:**

A systematic search was performed in the CINAHL, EMBASE, PsyInfo, MEDLINE, Cochrane Libraries, SCOPUS and Web of Science databases for English-language publications from January 2008 till October 2018 using a combination of MeSH-terms and keywords related to patient feedback, quality of health care, patient-centred care, program evaluation and public hospitals. The quality appraisal of the studies was conducted with the MMAT and the review protocol was published on PROSPERO. Narrative synthesis was used for evaluation of the effectiveness of the interventions on patient-centred quality of care.

**Results:**

Twenty papers reporting 20 studies met the inclusion criteria, of these, there was one cluster RCT, three before and after studies, four cross-sectional studies and 12 organisational case studies. In the quality appraisal, 11 studies were rated low, five medium and only two of high methodological quality. Two studies could not be appraised because insufficient information was provided. The papers reported on interventions to improve communication with patients, professional practices in continuity of care and care transitions, responsiveness to patients, patient education, the physical hospital environment, use of patient feedback by staff and on quality improvement projects. However, quantitative outcomes were only provided for interventions in the areas of communication, professional practices in continuity of care and care transitions and responsiveness to patients. Multi-component interventions which targeted both individual and organisational levels were more effective than single interventions. Outcome measures reported in the studies were patient experiences across various diverse dimensions including, communication, responsiveness, coordination of and access to care, or patient satisfaction with waiting times, physical environment and staff courtesy.

**Conclusion:**

Overall, it was found that there is limited evidence on the effectiveness of interventions, because few have been tested in well-designed trials, very few papers described the theoretical basis on which the intervention had been developed. Further research is needed to understand the choice and mechanism of action of the interventions used to improve patient experience.

## Background

**Fig. 1:**
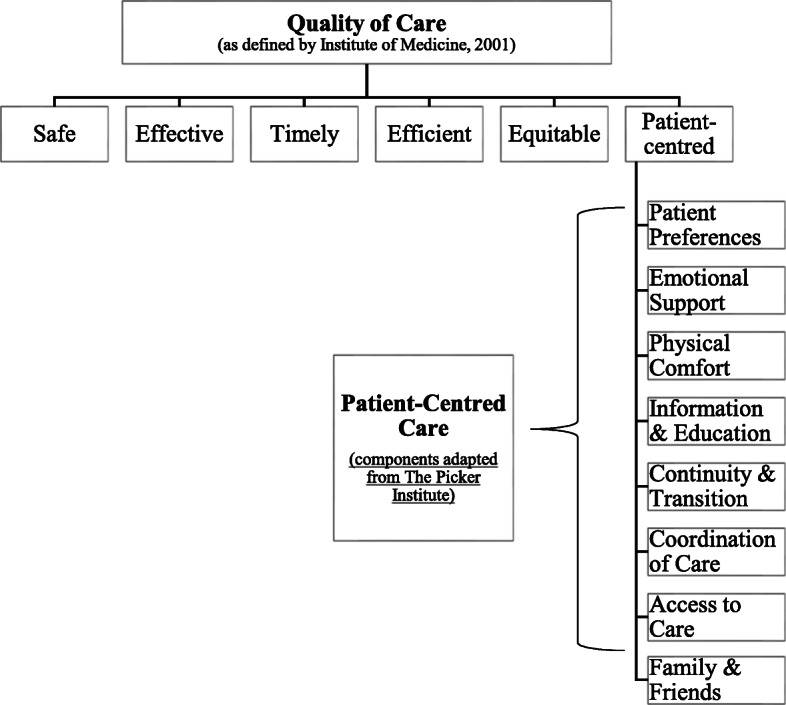
The relationship between quality of care and patient-centred care

Public health services have been moving towards putting patients at the centre of their care. Patient-centred care is defined as ‘care that is respectful of and responsive to individual patient preferences, needs and values, and ensuring that the patient’s values guide all clinical decisions [1]. Patient-centred care is considered to be one of the six domains of quality of care, where listening to and seeking to understand patients’ perspectives of their needs, is key to the delivery of good quality care [1]. For greater clarity, the relationship between quality of care and patient-centred care is illustrated in Fig. 1.

This approach to care (Fig. 1) promotes respect for patients’ preferences and values, and provision of emotional support, physical comfort, information, communication and education, continuity and transition of care, coordination of care, access to care and the involvement of family and friends [2–4]. These have been shown to be associated with clinical benefits [5–8] and healthcare cost reductions [9–11].

Patient-centred care is assessed by patient feedback of their experience often referred to as patient experience measures [8]. It is becoming common for these measures to be collected routinely in order to monitor patient-centred care [12–14] . The U.S. and U.K., were among the first to develop and implement nationally standardised surveys for measuring patients’ experiences. The American CAHPS (Consumer Assessment of Healthcare Providers and Systems) surveys were developed in 2008 and implemented in 2011. In the U.K., the reporting of the results of national standardised survey of NHS patients was made mandatory in their national health policy in 2010 [11, 15]. Other countries such as Australia, Canada, Denmark, the Netherlands also established systems for collecting patient experience measures under their health policies, in their efforts to improve patient-centred care and other domains of quality of care [12–14].

Although the various methods of collecting patient experience such as complaints, compliments, surveys, interviews and focus groups have been widely researched [16, 17], there is still a debate about the use of the terms ‘satisfaction’ and ‘experience’ in these measures, which are sometimes used interchangeably [18, 19]. Traditionally, up to the 1990s, patient satisfaction surveys were used to measure the quality of care from patients’ perspective. However there were theoretical arguments against its sensitivity and usefulness, as ‘satisfaction’ was conceptualised as people’s expectations and appraisals of care and not the actual ‘experience’ which relates to things that happened during care [19]. This led to the development of new patient experience surveys in the 2000s where the emphasis is on what happened to the patients during their hospital stay or clinic visit.

A recent systematic review [20], on the collection of patient experience and its use for quality improvements in health services, found that most quality improvement areas were in processes for admissions and producing patient education materials. Notably, these findings focused on areas that do not require changes to healthcare professionals’ behaviour, yet many components of the patient experience are integral to the interactions, patients have with healthcare professionals.

Moreover, findings (results) from patient experience surveys frequently highlight the lack of time in consultations, difficulties in understanding tests and information from doctors and lack of details and specificity from the survey needed for quality improvements [21–24]. The lack of patient involvement in developing quality improvement initiatives, the insufficient expertise by healthcare professionals in conducting improvement work and lack of time and resources were some of the key barriers to efforts to improve quality of care [7, 20, 25].

Understanding which interventions are effective in improving the various dimensions of patient-centred care is needed to achieve good quality care. Improvement efforts in health services cannot be made without the feedback of patients, participation or changes on the part of the healthcare professionals and the resources and support of their organisations [26, 27]. At present, it is unclear which interventions are effective and which behaviours need to change on the part of healthcare professionals and their organisations. The aim was to review the evidence about the impact of interventions informed by patient feedback on quality improvements in patient-centred care in hospital settings.

## Method

### Search strategies

The research adhered to the Preferred Reporting Items for Systematic Reviews and Meta-Analysis (PRISMA) 2009 checklist [28] and the review protocol was published (PROSPERO:CRD42018112964). The CINAHL, EMBASE, PsyInfo, MEDLINE, Cochrane Libraries, SCOPUS and Web of Science electronic databases were searched. Search terms included a combination of keywords, MeSH-terms and text words related to feedback OR patient satisfaction OR patient preference AND quality of health care OR outcome and process assessment (healthcare) OR outcome assessment (healthcare) OR treatment outcome OR process assessment (healthcare) OR program evaluation OR quality assurance, health care OR quality improvement OR quality indicators, healthcare OR standard of care OR patient safety OR patient-centred care OR healthcare quality OR quality of service OR health outcome AND hospital, public were entered. The search was limited to published studies from January 2008 as the literature documented the development of patient experience surveys in U.S. in 2008 and the reporting of standardised patient experience survey results in other countries from 2010.

### Study selection

#### Inclusion and exclusion criteria

Studies were included if they had investigated an adult population, reported feedback from patients and quality improvements to care, published in an English peer-reviewed journal from January 2008 till October 2018.

Using a standard form, information on study design, study setting, sample characteristics, sources of patient feedback, details of interventions used and outcomes were extracted by one author (EW) and verified by another author (JF). Where there was disagreement the third author (FM) reconciled the decision.

### Assessment of study quality

The Mixed Methods Appraisal Tool (MMAT) [29] was used to assess study quality. The MMAT includes specific criteria for mixed methods studies, as well as for qualitative and quantitative studies. In MMAT revised (2018), the authors discouraged the use of an overall numerical score to reflect the quality of the studies but to provide a detailed presentation of the ratings of the criteria to reflect the quality of the included studies [30]. The assessment is made against five criteria, scored as ‘Yes,’ ‘No’ or ‘Can’t tell’, and it was developed systematically [31]. For ease of discussion, in this review the studies were ranked as high (all criteria met), medium (four out of five criteria met) and low (three or less criteria met).

### Data synthesis and analysis

Data synthesis allows researchers to critique and integrate research data from diverse disciplinary perspectives and studies which have used qualitative, quantitative, and mixed designs. Studies with multiple components intervention were coded to each of the intervention areas identified and according to the quality of the study; leading to some being counted more than once in the summary table. This approach is recommended for reviews seeking to understand the effectiveness of certain intervention areas, by categorising interventions by commonalities rather than considering the multiple components intervention as a whole unit [32, 33]. Finally, a narrative synthesis was used to report the evaluation of the studies.

## Results

### Search results

**Fig. 2:**
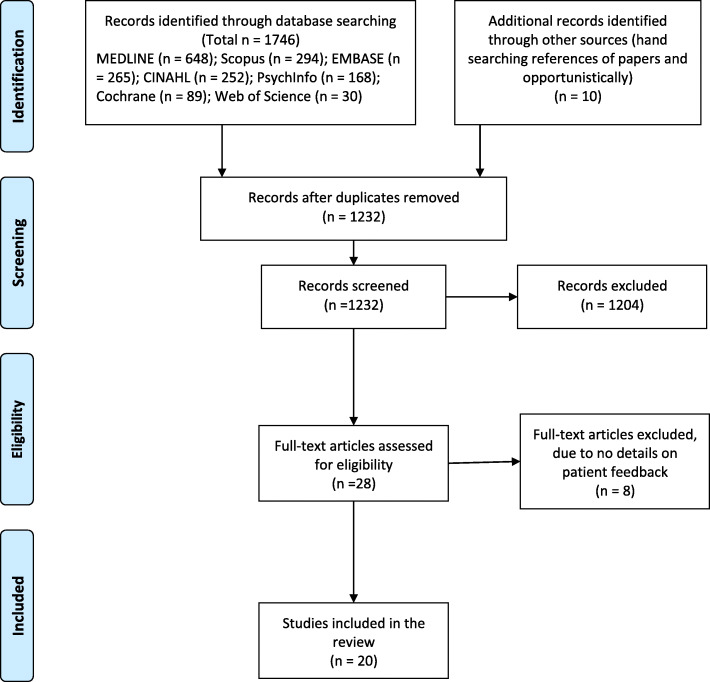
PRISMA flowchart showing the selection of studies

The initial search returned 1746 papers (Fig. 2), which were imported to Endnote and subsequently to Covidence [34] for screening; after removing duplicates, 1232 papers were retained. The title and abstracts were screened against the inclusion criteria. Two authors (EW and JF or EW and FM) assessed the papers and yielded 28 papers for inclusion. The final retention of 20 papers were made by consensus, any disagreements were resolved by consensus or consultation with a third author (FM or JF). The main reason for exclusion at this stage was that papers mentioned inclusion of patient feedback in the abstract but did not give any details of the patient feedback collected.

### Quality assessment

Assessment of studies using MMAT indicated that two studies rated high fulfilling all five criteria, five studies rated medium meeting four criteria, 11 studies met only three or fewer criteria were rated as low and two studies could not be appraised because details regarding research aims, data collection methods or analysis were not provided (See Additional file 1).

### Methodological characteristics and main findings

All studies included in the final review were based in hospital settings, and of these, three also included the health services’ primary and community care settings as they formed part of the organisation. The methodological characteristics and main findings are summarised in Table 1.

**Table 1:** Methodological characteristics and main findings

First author (year); Country; Setting	Interventions	Study characteristics; design; method; data source	Participants’ characteristics	Main results	Quality Assessment
RCT studies					
Reeves (2013) [35]; UK; 2 NHS Trusts	The interventions for 3 groups: 1) Control group: CQC survey results given to Director of Nursing. 2) Basic feedback group: Individual letters with detailed ward-level CQC survey, results sent to nurses and their matrons. 3) Feedback Plus group: Same as Basic feedback group with the addition of ward meetings with study researchers to discuss CQC survey results and plan improvements in practice.	‘Pilot study’ for cluster RCT; NHS Care Quality Commission (CQC’s) Inpatient Questionnaire-subset nursing care with 20 questions scores ranged between 0 to 100.	4236/9565 patients surveyed across 18 wards (47% response rate). The 3 groups consisted of 6 wards, (No. of nurses in the wards were not reported)	The mean score was 75.4 at baseline. Feedback Plus wards experienced an improvement in scores the difference between Control and Feedback Plus wards is 8.28 ± 7.2 (*p* = 0.02). There is no evidence that Basic feedback group lead to improved patient experiences, or that nurse pay attention to results when they are in printed form.	Medium
Before-After Studies/ Cross-sectional Studies					
Harnett (2010) [36]; US; 1 hospital (Pre-operative clinic)	The interventions were: 1) Provide anaesthesia education programme to Nurse Practitioners and after the training, all assessments for a single patient was conducted by a Nurse practitioner with laboratory technician conducting tests in the same room at the same visit. 2) Change in Nurse Practitioner shifts from 8 to 10 h to improve room utilisation. 3) Blank appointment slots were left for surgical add-ons instead of disrupting already scheduled patients. 4) Postcard appointment reminder sent to patients in advance. 5) 2 h weekly staff meetings for clinical and non-clinical staff on customer service, patient relations, and teamwork.	Before – after study; study specific 14- item questionnaire (Likert scale 1–5) consisting of satisfaction with clinical providers and with organisational aspects of clinic visit was administered to different patients who attended the preoperative clinic at two time periods (March 2005 and March 2006).	872/1100 patients responded (79% response rate), with 443 patients in cycle 1 and 429 patients in cycle 2.	The questionnaire scores for 3 out of 14 items showed significant improvement (*P* ≤ 0.01) The 3 items related to the explanation of the preoperative clinic by the surgeon’s office, courtesy and efficiency of the clinic staff and satisfaction with the amount of waiting time. The average waiting times reduced from 92 ± 10 mins to 42 ± 5 mins.	Medium
Aboumatar (2015) [37]; US; 52 Hospitals	No intervention	Before - after study; hospital performance in the HCAHP survey was extracted from the publicly available December 2012 HCAHP report; study specific online survey of a set of 12 binary response questions and 3 open-ended questions were emailed to participants who were nominated by their hospital CEOs.	52/169 hospitals recruited based on the study’s high-performance criteria for at least 1 HCAHPS domain; 138 respondents from 52 hospitals participated in the survey.	High performing hospitals reported use of interventions on both the patient and system levels. *Patient level interventions* 1) Improve responsiveness to patient; 83% used proactive nursing round; 62% used executive/leader rounds. 2) Discharge experience; 56% used multidisciplinary rounds; 54% used post discharged calls; 52% used discharge folders. 3) Patient-clinician interactions; 65% promoted specific desired behaviours; 60% set behavioural standards where employees were held accountable. *System-level* 4) Engage and educate employees (71%) and leaders (83%) about the behaviours needed to ensure positive patient experiences. 5) Hospital leaders monitored and audited desired behaviours to hold employees accountable (50%).	Medium
Buurman (2016) [38]; The Netherlands; 1 hospital	The implementations were: 1) Education of interns, residents, staff. 2) Medical interns given targets to issue PPDL. 3) Standardised content & templates. 4) Integrating PPDL into electronic medical record. 5) Integrating PPDL into hospital wide policy.	Before – after study; structured telephone interviews with patients, 1 week after discharge was conducted by a research nurse; focus group conducted with nurses and physicians on the use of personalised patient discharge letter (PPDL) in daily practice.	141 patients participated in this study. 111 patients participated in the pre-implementation phase and 30 patients in the post implementation phase. Participants for focus groups (not reported).	Patient satisfaction with the PPDL was 7.3 out of 10. The level of implementation increased from 30 to 51% because of incorporating the PPDL into the electronic patient record (EPR) and professional education.	Low
Kleefstra (2016) [39]; The Netherlands; 10 health inspectors	Provide negative patient reviews on hospital rating sites on a hospital that was supervised by the health inspector (participant)	Before-after study; Semi-structured interviews were conducted with the participants, subsequently negative patient reviews on hospital rating sites and the hospital contextual details were emailed to the participants and they were interviewed again 4–6 months later.	10 Senior Health inspectors	23% of patient reviews were deemed relevant for risk identification by the senior health inspectors. The reviews which included major safety problems, severe damage or consequences for the patient and structural organisation problems, malfunction of doctor was deemed relevant.	Low
Ancarani (2009) [40]; Italy; 7 hospitals	No intervention	Cross-sectional study; study specific organisational climate survey was administered once to all medical staff and the SERVQUAL instrument measuring patient satisfaction was administered once to all patients in 47 wards in 7 public hospitals. All members of the medical staff and consecutive patients prior to discharge were also interviewed.	625 Healthcare professionals (470 nurses and 155 physicians) and 1018 patients participate in the study.	Organisational model stressing openness, change and innovation and organisational model emphasizing cohesion and workers’ morale are positively related to patient satisfaction, while a model based on managerial control is negatively associated with patient satisfaction.	Medium
Richard (2010) [41]; Canada; 1 hospital cancer centre	No intervention	Cross-sectional study; study specific survey using 21 items from a Canadian validated question bank measuring patient satisfaction was administered over 1-month period to ambulatory cancer patients.	276/575 patients responded (48% response rate).	It was reported that wait times and telephone contact with healthcare providers were the 2 areas of lowest satisfaction. 72.5% (*n* = 103) of patients followed by a nurse navigator; were satisfied with the length of time spent in the waiting room compared with 66.2% for patients without a nurse navigator (*n* = 77).	Low
Madden (2010) [42]; UK; NHS trusts	No intervention	Secondary data analysis from two national surveys of patient experiences in 2000 and 2004 and Thames Cancer Registry. The respondents from the national surveys of patient experience were surveyed at different times after discharge and a year elapse between data collection and reporting. The cancer registry contains area registration of patients in South East England, their diagnosis and clinical information from hospitals.	69,660 patients responded; 65,337/88293 patients from 172 hospital trusts responded (74% response rate) in year 2000 and 4323/7860 patients from 49 hospital trusts responded (55% response rate) in year 2004.	Comparison between 2000 and 2004 surveys showed some overall national improvements in areas of information, communication and trust in health professionals. Only breast cancer patients from 3 health trusts were compared due to data availability and there is a significant decline in 2 areas; ‘ease of understanding of tests from doctors’ and ‘feeling confidence in the doctor at the last outpatient appointment’.	Low
Case Studies and participatory action studies					
Reeves (2008) [43]; UK; 24 NHS trusts	No intervention	Case series; semi-structured interviews using interview guide specific to the study was conducted with patient survey leads from 24 NHS trusts.	24 patient survey leads who held varied positions such as Director of Nursing, Director of Patient and Public Involvement, Quality Development Manager and Head of Clinical Governance were interviewed.	Actions implemented for quality improvement were: 1) Action plans aimed at improving the quality of care and for measuring the success of those plans. 2) Implementation of action plans was now part of some individuals’ performance assessment. 3) Asking patients to keep records of the source of disturbing noises. 4) Floor coverings were changed, quieter waste bins. 5) were installed, and, where possible, patients admitted overnight were put into a separate area. 6) produced comprehensive discharge information packs, which were given to patients on admission. Barriers identified: 1) Difficulty engaging clinicians because survey findings were not sufficiently specific to specialties, departments or wards. 2) Culture of the organisation. 3) Lack of knowledge of effective interventions. 4) Lack of statistical expertise. 5) Limited time and resources.	Medium
Long (2008) [44]; Australia; 1 hospital	No intervention	Case study; study reported a four-phase methodology; Phase 1, the conduct of discovery interviews to identify and develop quality improvement strategies; Phase 2, strategies were sent back to the same participants for validation; Phase 3, focus group conducted with clinicians and quality managers to validate the quality improvement strategies identified and phase 4 integrating the improvement strategies with the hospital’s quality improvement programme.	30 patients who has experience an adverse event and six quality managers and clinicians.	The improvement areas identified and validated are in communication with consumers, consumers education, assessment and prevention of adverse events and clinical environment contributing to the occurrence of adverse events.	Low
Hsieh (2010) [45]; Taiwan; 1 Teaching hospital	No intervention	Case study; study specific critical incident questionnaire was employed for all complainants over 3 months by hospital social workers trained in critical incident technique and non-participant observation of the hospital was conducted over a-3-month period by researcher.	59 complainants completed the critical incident questionnaire.	The most common themes identified for cause of complaints were care/treatment, humaneness and communication. The study found that of 149 resolutions, 105 taken by the hospital involved an explanation of the facts to complainants (*n* = 41), investigation of events (*n* = 33) and empathy with complainants (*n* = 31). The lack of any systematic use of complaints data was reported as a failure for the hospital.	Medium
Latta (2010) [46]; Australia;1 Health service with 7 public and private hospitals.	No intervention	Case study (No details reported)	None reported	Reported the implementation of integrated case management and care pathway had led to improved risk management, reduced lengths of stays, healthcare costs, and increased patient and staff satisfaction.	Low
Schneider (2010) [47]; South Africa; 1 public hospital	No intervention	Case study; observations and informal conversations with patients and staff in emergency department, admission ward and medical wards were conducted. Interviews were conducted with 30 staff and on the spot, surveys conducted with 41 patients while they are waiting in the emergency department and 2 focus groups conducted.	71 participants consisted of 30 hospital staff and 41 patients. Focus groups participants (not reported).	It was reported that patient’s actions were oriented to two main goals: obtaining care and preserving their sense of self and dignity.	Medium
Davies (2011) [48]; US; Veterans hospitals	No intervention	Case study; selection of hospitals was based on their stable high or low scores on the dimension of emotional support derived from the Survey of Healthcare Experiences of Patients (SHEP) conducted from 2002 to 2006; semi-structured interviews was conducted by telephone with respondents at work.	8 healthcare professionals consisted of 2, executive directors, 2 patient advocates, 2 customer service managers 1 ward nurse and 1 advanced nurse practitioner from 2 Veterans hospital were interviewed in the study.	Promoters of quality improvements found: 1) Developing patient-centred cultures 2) quality improvement structures such as regular data review 3) Training staff in patient-centred behaviours. 4) The influence of incentives 5) The role of 6) nursing leadership 7) Triangulating survey data with other data on patients’ views Barriers of quality improvements found: 1) Clinical Scepticisms 2) Defensiveness and resistance to change 3) Lack of staff selection, training or support 4) Lack of timely feedback 5) Lack of specificity and discrimination of survey results 6) Uncertainty about effective interventions	High
Hsieh (2011) [49]; Taiwan; 1 hospital	No intervention	Case study; semi-structured interviews were conducted with hospital staff, government staff and non-government staff. Administered semi-structured study specific questionnaires for hospital staff and review of documentation of activities in the hospital. A separate study specific critical incident questionnaire was employed for all complainants over 3 months and non-participant observation of the hospital was conducted over a 3-month period.	123 participants consisted of 4 key managers and social workers, 4 government staff, 3 non-government staff, 53/72 respondents to the questionnaire (73.6% response rate) and 59 complainants completed the critical incident questionnaire.	This study revealed that the hospital attempted to resolve complaints on a case-by-case basis. It did not act on these complaints as a collective group to identify systemic problems and deficiencies.	Medium
Piper (2012) [50]; Australia; 7 hospitals	Experience-based co-design (EBCD) programme using a five-phase methodology within 43 to 44.5 weeks’ timeframe.	Case study; selection of 7 hospitals based on their previous participation in the EBCD programme. Documentation from the EBCD programme provided by the 7 hospital and semi-structured interviews with staff and consumers.	117 participants consisted of 3 department staff, 59 frontline staff & management, 41 project staff and 26 consumers.	EBCD were used in improvement areas of: 1) Patient and carer comfort 2) Physical spaces 3) Respect and courtesy, information for patients and patient perceptions It was reported to have improve operational efficacy and inter-person dynamics of care. Main barriers to the use of EBCD identified were: 1) Sustaining consumer engagement from ambulant population in emergency departments. 2) Tailoring to consumer preferences & constraints. 3) Perceived as separate & additional task.	High
Tsianakas (2012) [51, 52]; UK; 1 Cancer centre	Experience-based co-design project over 12 months	Participatory action research; fieldwork involved 36 filmed narrative patient interviews, 219 h of participant observation of clinical areas along the patient pathway and 63 staff interviews and facilitated a co-design change process with patient and staff participants. 4 staff and 5 patients were interviewed again about their views on the value of the approach and its key characteristics.	99 participants consisted of 36 (23 breast and 13 lung cancer) patients and 63 staff.	It was reported patients living with breast and lung cancer identified similar issues in receiving diagnosis, continuity of care, communications between staff and patients, appointments process and inpatient experience that shaped their experience.	High
McDowell (2013) [53]; UK; 3 NHS Trusts	No intervention	Case study	None reported	Described the implementation of an engagement model of both patients and staff encourages ownership and co-creation of solutions.	Low
Abuhejleh (2016) [54]; UAE; 1 Hospital	Use of Lean six sigma methodology and Kaizen Plan-Do-Check-Act cycles	Case study; interviews were conducted in the hospital and the information collected from the interviewees was reviewed and verified by a LEAN project leader at the hospital.	No details reported	The innovation projects reported decreased in patient access and waiting time, improved safety and patient satisfaction and supported the hospital culture of empowering front-line caregivers.	Low
Blackwell (2017) [55]; UK; 1 Hospital	Experience-based co-design project over 19 months.	Participatory action research;150 h of non-participant observations, semi-structured interviews with 15 staff members about their experiences of palliative care delivery, 5 focus groups with 64 staff members to explore challenges in delivering palliative care, 10 filmed semi-structured interviews with palliative care patients or their family members and 1 co-design event with staff, patients and family members.	93 participants consisted of 79 staff, 10 patients & caregivers and 14 staff, patients and facilitators.	The study identified quality improvement priorities leading to changes in Emergency Department-palliative care processes. It also led to the creation of a patient-family-staff experience training DVD to encourage application of generic design principles for improving palliative care in the emergency department.	High

#### Study location, sample and design

The studies were conducted in eight countries, UK (*n* = 6), Australia (*n* = 3), US (n = 3), the Netherlands (*n* = 2), Taiwan (*n* = 2), Canada (*n* = 1), Italy (*n* = 1), South Africa (*n* = 1) and the United Arab Emirates (*n* = 1). The different sources of feedback in the studies were interviews (*n* = 7), patient experience surveys (*n* = 6), patients’ narratives of their experience (*n* = 2), complaints (*n* = 2), patients’ perception of service quality (*n* = 1), patient views on access (*n* = 1) and patient ratings online of hospitals (*n* = 1).

A total of 77,588 participants contributed data to 17 studies, and participants characteristics were not reported in three [46, 53, 54]. The 20 studies were conducted in inpatient or outpatient settings in public hospitals with five studies providing additional details on the speciality settings. They included specialised cancer treatment (*n* = 3) and emergency medicine (*n* = 2).

The 20 studies comprised a cluster randomised control trial (*n* = 1), before and after studies (*n* = 3), cross-sectional studies (*n* = 4), and organisational case studies (*n* = 12). The outcome measures in all the studies were on patient experience or patient satisfaction with waiting times, physical environment and courtesy of staff, which are components of the patient experience.

### Intervention

#### Areas of interventions

The interventions proposed and implemented in the studies were synthesized according to the target area of the interventions. Multi-component interventions targeting more than one area are accounted for in each of their target area of intervention, to provide a comprehensive view of intervention areas. Further details on the nature and examples of interventions in the areas of communication with patients, professional practices, clinicians’ responsiveness to patients, patient education, the physical hospital environment, quality improvements, and improving the use of feedback are provided in Table 2. Only one of the studies [40] reported their theoretical basis and four studies [50, 51, 54, 55] specified the use of quality improvement and experience-based co-design methodology.

**Table 2:** Areas of intervention from the included studies

Target areas of intervention	No. of studies & Quality assessment^a^				Nature and examples of the interventions
	H	M	L	NA	
Communication [39, 42–44, 47, 67, 68, 70] (*n* = 8)	2	1	5		Using slogans and acronyms to promote communication, interpersonal skills training for staff, set behavioural standards for staff and use of filmed patient and family experience interviews as communication education.
Professional practices (Continuity of care and care transitions) [37, 38, 44, 46, 47, 52, 55] (*n* = 7)	2		5		Reduce repetitive assessments by multiple staff, plan for diagnosis giving in a dedicated space and provide written and verbal discharge information to patients,
Responsiveness to patient (Respect for preferences and emotional support) [37, 41, 42, 45, 48, 50] (*n* = 6)		1	5		Introduction of hourly proactive nursing rounds and weekly senior executive rounds, provide telephone contact to nurses regarding health concerns and clinical leads to review information flow about patient care along the care pathway.
Patient education [42–44, 50] (*n* = 4)		1	3		Provide information pack and handouts on treatment options, care navigation and discharge processes to patient and families.
The physical environment [43, 50] (*n* = 2)		1	1		Made changes to floor coverings to reduce noise, creation of family rooms or quiet spaces in the hospitals
Improve use of feedback [35, 54] (*n* = 2)		1		1	Schedule meetings to discuss patient experience results and plan improvements and triangulate multiple sources of data to understand the feedback.
Quality improvement [43, 48] (*n* = 3)		2		1	Provide structure and support by the organisation for the identification and implementation of quality improvements and monitor quality improvements as part of staff performance assessment.

^a^*H* high, *M* medium, *L* low, *NA* not accessed

#### Communication

Interpersonal communications about health conditions and care transitions between patients and staff were the key area of intervention identified in improving patient experience in the studies in this review. The interventions targeted changes in staff’s communication behaviours, for example, provision of weekly education sessions on communication skills and setting behavioural targets for staff. The frequency and mode of delivery of the education sessions reported were varied but they shared similar education content on customer service and interpersonal communication skills [36–38]. However, significant increase in satisfaction with explanation given and courtesy and efficiency of staff was only reported in Harnett et al.’s study [36] where the education component is part of a suite of other interventions.

In addition to staff education, two studies [37, 38] also reported on organisational level interventions as part of the suite of intervention. Aboumater et al. [37] observed that hospitals with high patient experience scores promoted specific behaviours on communication and engagement of patients to staff using acronyms and slogans on (65%) and set standards and targets for staff for patient-centre and excellent service (60%). This observation is also noted by Buurman and colleagues [38] in their study where targets were set for staff, adoption rates of personalised communication with patients on discharge increased by 20% over 3 years. However, these changes cannot be assumed to be related to the interventions in the absence of a control group, in their study designs, it could be attributed to the passage of time or other factors.

Two further studies [51, 55] used experience-based co-design as an approach to engage, seek patient feedback on their experiences and views to identify improvements, discuss, design a suite of changes in communication, and professional practices. As the experience-based co-design methodology in its nature is about tailoring to the context, the findings from these studies may be limited to the experience of patients accessing cancer treatment services and emergency departments of hospitals. There was no measurement of patient experience, but the patients reported having had good experience when interviewed about the effects of the changes.

#### Professional practices in continuity of care and care transitions

Four studies highlighted discharge planning and associated care processes such as follow-up phone contact, giving written discharge information to patients as a focus area in improving patient experience. It was found in two studies that use of both individual and organisational level interventions was significantly more likely to have a difference in patient experience. Aboumater and colleagues [37] reported that 52–56% of hospitals with high patient experience survey scores, indicative of high quality hospitalisation experience in their study, implemented multi-disciplinary rounds, follow-up with patients via phone calls post-discharge by nurses and used discharge folders for information sharing and consolidation. Organisational level interventions of using templates for personalised discharge letters, incorporating personalised discharge letters into the computer system of electronic medical records and integration of its use as hospital-wide policy were associated with an increase in the use of personalised discharge letter from 30 to 50% in the hospital over a 3 year period in Buurman et al.’s study [38]. Furthermore, two case studies [51, 55] that provided an in-depth understanding of the experience-based co-design approach supports this observation between intervention to care processes and good patient experience. These studies explore the experience-based co-design approach in the redesign of palliative care and cancer care processes as part of a suite of interventions, where good experience was reported by interviewed patients.

#### Responsiveness to patient

The role of nurses was highlighted as a common component of the interventions employed in three studies [37, 41, 48], to improve the patient experience. The interventions targeted behaviours that were perceived by patients as respectful, courteous, prompt and person-centred. However, only weak associations between these interventions and positive patient experience were reported. In Abounmater et al. which used proactive nursing rounds (83%), and executives and leaders making rounds to engage and respond to patients (62%) [37], had high patient experience scores. Richard et al.’s cross-sectional study [41] observed that patients with nurse navigator support compared to those without reported higher satisfaction with waiting times.

The role of doctors was generally not explored with the exception of Madden and Davis’ study [42] where secondary data analysis was conducted to compare the results of two national patient experience surveys conducted in 2000 and 2004. It is interesting to note that this is the only study that reported a downward trend in aspects of patient experience with doctors (confidence in doctor and understanding of tests from doctors’ explanation) for patients using breast cancer services in three health services. This was in spite of reported upward trend on a national level (across 172 health trusts in UK). The influences on this downtrend trend is unknown as there were no reported investigation on the probable causes or associations.

#### Patient education

Conceptualisation of patient education differed among studies. In Reeves and Seccombe’s study [43], patients were given a comprehensive patient information pack about the discharge processes. This intervention was further complemented with the organisational level intervention of inclusion of its implementation action plans as part of staff performance assessment. While two other studies [42, 50] did not provide details and defined it as information for patients. There was no significant evidence on any association or efficacy of interventions in this area from these studies.

#### The physical environment

Interventions to improve the physical environment found in two studies, focused on engaging patients in the redesign of physical spaces in the emergency department [50] and reduction of noise levels in the hospital [43]. Overall, the changes in the physical environment could not be solely associated with changes to the patient experience, as these interventions were part of a larger suite of interventions.

#### Improve use of feedback

Reeves and West’s study was the only cluster RCT [35] in this review. They found significantly better experience survey scores among patients in the condition where wards held facilitated meetings to review patient feedback and plan improvements compared to the two other conditions (feedback sent to the Matron of ward and feedback on ward level sent to individual nurses). From the findings of the study, the authors hypothesised that it is the opportunity for facilitated delivery of the feedback to nurses that increased the acceptability of the feedback that prompted the change in behaviour.

#### Quality improvement

The studies [43, 48] that investigated interventions used in quality improvement projects suggested that it is necessary to approach this at both the staff and organisational levels. They observed that good patient experience was possible when there was regular data review, effective implementation of action plans, and incentives and staff performance review by their organisations.

## Discussion

The results of this review show that interventions employed in the included studies, predominantly target and support the theoretical dimensions of patient-centred care. Interpersonal communication between healthcare professionals and patients about their health conditions and care, processes affecting care continuity and discharge planning and showing respect for patient preferences and providing emotional support clearly emerged as important intervention areas, most frequently noted in the 20 studies. However, the efficacy of the interventions must be interpreted with caution because causal relationships were mostly not tested in the studies included in this review.

### Strengths and limitations

The strength of this review is the specific focus and inclusion of the use of patient feedback for improving patient-centred care in the search strategy for the review. The search strategy was designed in consultation with an information analyst, to produce a replicable search for all relevant multiple databases, using MeSH search terms and the inclusion of all study designs, single and multiple interventions and variety of countries, to provide a search of the evidence that has been applied to the existing context in health services rather than just research settings.

We acknowledged some limitations in this review, only studies published in English language and after January 2008 were included. There could be other relevant studies published prior and in other languages that were missed. Further details on the interventions in the included studies could also be missed as no further contact was made with their respective authors.

### Main findings

There are several possible explanations for this weak body of evidence on the efficacy of the various interventions, firstly, the study designs employed in the studies were mainly correlational and qualitative and secondly, the quality of the studies. There is only one cluster RCT in this review that provided evidence that patient feedback was effective in improving quality of care when it was facilitated and discussed with nurses and planned for at ward level compared to other conditions where it was not facilitated or discussed. Overall, 11 studies reported improvement in patient experience outcomes, but only five studies quantified their findings by reporting on the changes in outcome measures.

The quality of evidence of the five quantitative studies that reported outcome measures was low, beyond the limited representativeness of the study populations in some of the studies, the weak associations between the interventions and outcomes with no acknowledgment of potential confounders such as the passage of time.

The qualitative studies in this review were more varied in study quality, four of the studies were conducted well with detailed reporting. The studies highlighted how experience-based co-design methodology was utilised in acute care settings to engage and partner patients in making improvements to care and also contribute to the understanding of the areas of care that were deemed important by patients.

Studies that used multiple interventions targeting change on both individual and organisational levels were associated with better outcomes than those studies with single interventions. This review found that interpersonal communication training for healthcare professionals combined with organisational policies of setting targets and promoting behavioural standards for the staff were associated with improved (increased) patient experience. Similarly, this association was also found with implementing processes and practices with multidisciplinary team meetings and sharing of discharge information practices, in conjunction with organisational policies of setting targets and promoting behavioural standards for the staff.

These findings are in line with studies [56, 57] that explored a system view in implementing interventions where considerations are given to mediating factors organised by structure (organisational characteristics), process (care processes) and outcome (patient experience, clinical outcomes) using Donabedian’s model. This is further supported by findings from studies [58, 59] investigating factors needed for successful implementation and integration of interventions to routine work using the Normalisation Process Theory [60, 61]. With the acknowledgement of targeting change on multiple levels within a system, it is not surprising that there is a growing body of literature on developing and evaluating multiple components interventions [62].

Beyond the limitations of the study designs and quality of the studies, a plausible explanation for the weak evidence is the lack of explicit use of theory in the intervention development or discussion of results in the majority of the studies. The importance of using theory is reflected in the growing research of using behavioural and organisational theories in the design of interventions involving professional practice and the understanding of which mechanism or elements of the interventions are the most important [63]. For example, in the studies targeting improving communications between patients and healthcare professionals, the effective interventions were using a combination of educational sessions for staff and action planning and monitoring interventions by organisations. Without being explicit about their theory of change, in the communication behaviours in those studies, it is plausible that educational sessions for staff were conducted to engage staff on communication as a priority, instead of their lack of skills. If that was true, more targeted interventions to address engagement and prioritisation by healthcare professionals could be more effective.

There are different theories that may be relevant for developing interventions at multiple levels, using approaches that address, cognitive, educational and organisational theories that can contribute to changing healthcare professionals’ behaviours [64]. For example, theories such as theory of planned behaviour and social learning theory [65–67] may be more relevant to interventions directed at individuals and teams. On the other hand, organisational theories such as Continuous Quality Improvement [68] and organisational quality culture [69] may be more relevant to interventions directed at service redesign for the whole hospital with multiple stakeholders [63].

### Further research

From the review findings, the field of research could explore the gap in the explicit use of theory in their target for change and choice of interventions. This will enable the comparison of interventions and their mechanism of action, across settings to build the evidence base. Beyond those interventions found in this review, another gap to address is the lack of research in the interventions targeting the emotional support, access to care, involvement of family and friends dimensions of patient-centred care. It could provide further insights in the interpersonal relationship between patients, their family and the healthcare professional and its impact on patient-centred care.

There is also room for further progress in examining the acceptance and utilisation of patient experience in the development and evaluation of improvement efforts in patient-centred care. Despite the widely acknowledged concept of patient-centred care, the low number of studies found in this review that includes patients’ perspective and experiences of care is professionally and practically concerning.

The conceptual definitions and differentiation between patient experience and satisfaction are still debated in the existing literature [19, 70, 71]. However, in the reviewed studies the authors did not differentiate between these concepts. In some studies in the measurement of patient satisfaction, the focus was on the experience of the process and feeling, rather than the concept of satisfaction where their expectations are met or not. In other studies on patient experience measurement, the focus was on the patients’ expectations. The lack of conceptual differentiation of these concepts could be addressed in future studies as there are implications in their operationalisation and comparability of findings.

### Implications for practice

The evidence from the reviewed studies suggests that health services are collecting feedback from patients on their experience either locally or through nationally standardised survey instruments and increasingly reporting them as one of their performance indicators. Not surprisingly, the collection and reporting of patient experience in itself, does not improve care. Considering the evidence from the review, the patient experience collected needs to be discussed and facilitated with healthcare professionals in their respective operational units in order to provide opportunities for them to engage and act on the feedback to improve care.

The finding on the strong focus on interventions targeting communication between healthcare professionals and patients suggests that communication is akin to the ‘delivery’ system for the dimensions of patient-centred care. This could be a consideration for health services as a starting point as it has also been recommended as an area of focus with good cost-benefit to health services [72].

## Conclusion

This review shows that incorporating patient feedback of their experience into research on quality patient-centred care is still an emerging field. The limitations outlined show that the degree of effectiveness attached to the different interventions must be interpreted with caution. However, the findings of this review can inform researchers, healthcare professionals, health systems and policy makers to focus on interventions, practice guidelines and strategies that incorporates patient feedback of their experience in patient-centred care improvement work. Care is truly patient-centred when it is guided by the perspective of the one that matters - the patient.

## Supplementary information

**Additional file 1.** Risk of bias assessment.

## Data Availability

The data generated or analysed during this study available from corresponding author on reasonable request.

## Abbreviations

CAHPS: Consumer Assessment of Healthcare Providers and Systems
NHS: National Health Service
PRISMA: Preferred Reporting Items for Systematic Reviews and Meta-Analysis
MMAT: Mixed Methods Appraisal Tool
RCT: Randomized controlled trial
